# Hidden in Plain Sight: Delayed Diagnosis of Poorly Cohesive Gastric Carcinoma in a Young Male Patient

**DOI:** 10.7759/cureus.87097

**Published:** 2025-07-01

**Authors:** Tyler R Ellett, Andrew Pippas, Humberto Rios, William F Willett, Maria B Diaz

**Affiliations:** 1 Internal Medicine, St. Francis Emory Healthcare, Columbus, USA; 2 Hematology and Oncology, Piedmont Healthcare, Columbus, USA; 3 Gastroenterology and Hepatology, St. Francis Emory Healthcare, Columbus, USA; 4 Pathology, St. Francis Emory Healthcare, Columbus, USA; 5 Internal Medicine, Mercer University School of Medicine, Columbus, USA

**Keywords:** adenocarcinoma-gastric type, diffuse type, early gastric cancer (egc), early-onset cancer, helicobacter pylori, h.pylori

## Abstract

Poorly cohesive gastric adenocarcinoma is classified as a diffuse type of gastrointestinal tract tumor composed of undifferentiated cells with an inherent propensity for aggressive behavior. Recent trends in surveillance indicate that gastric cancer (GC) is affecting younger adults and is distinct from GC that occurs later in life. Early-onset gastric cancer (EOGC) is associated with various environmental and genetic factors. Younger patients with recurrent pyrosis may warrant earlier surveillance in light of this worrisome trend. Herein, we discuss a young male patient who developed non-hereditary, poorly cohesive gastric adenocarcinoma with an extended history of pyrosis despite long-term acid suppression and triple therapy for *Helicobacter pylori *(*H. pylori*).

## Introduction

Despite significant advancements, gastric cancer (GC) remains the fifth most common cancer and the fifth leading cause of cancer-related death [1]. In the United States, 30% of new GC cases occur in young adults, with a strong association to diffuse-type histology, higher grade, and metastatic disease [2]. The mechanisms for this are still poorly understood. Known environmental risk factors appear less prominent when compared to later-onset GC, and reduced lifetime exposure suggests a hereditary component. Heritable GC accounts for a subset of early-onset cases, with approximately 77% of GC cases being sporadic [3]. Early-onset GC (EOGC), defined as occurring before age 50, is linked to distinct mutations in the BANP, MUC5B, and RHOA genes, epigenetic alterations, and dysbiosis caused by *Helicobacter pylori* (*H. pylori*) eradication therapy [4]. Common presenting symptoms include nausea, vomiting, and bloating. Due to a lack of routine or standardized screening, it is often overlooked, leading to a delayed diagnosis.

## Case presentation

**Figure 1:**
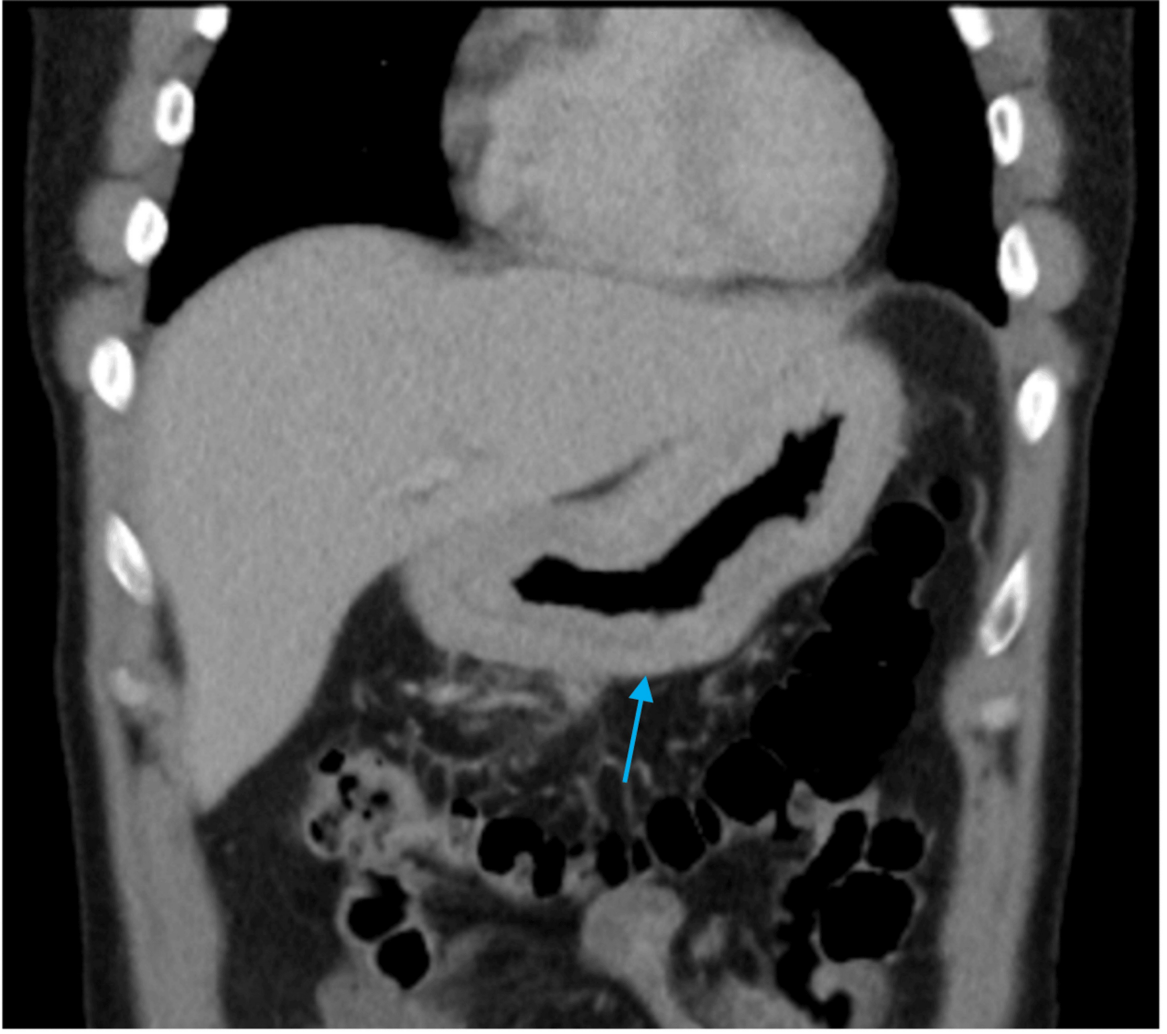
Computed tomography of the abdomen with findings of diffuse gastric wall thickening (blue arrow).

A previously healthy 41-year-old Nigerian male patient presented to the emergency department with a chief complaint of discomfort in the stomach with associated bloating and diarrhea. The initial onset of abdominal pain began in 1999, with subsequent visits for similar discomfort in 2009, 2015, and 2019. He received treatment with cimetidine, omeprazole, and triple therapy for *H. pylori *infection based on clinical suspicion. No testing for confirmation or eradication was performed. He had no prior medical history, surgeries, or medications. His social history was insignificant, and his family history was negative for gastrointestinal disease. Complete blood count and comprehensive metabolic panel were normal. Computed tomography (CT) of the chest, abdomen, and pelvis revealed diffuse gastric wall thickening with heterogeneously enhancing necrotic lymph nodes in the mesentery (Figure 1). Esophagogastroduodenoscopy (EGD) revealed decreased distensibility in the stomach with an ulcerated lesion in the antrum. The pylorus had a linitis plastica-like appearance, and multiple ulcerations with a broad base were noted above the tumoral lesion.

**Figure 2:**
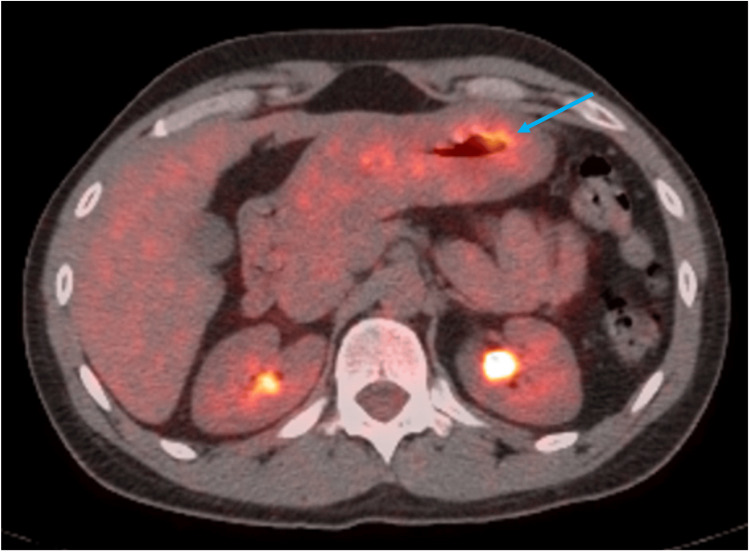
Positron emission tomography with abnormal uptake of fluorodeoxyglucose in the mid- to distal gastric body (blue arrow).

**Figure 3:**
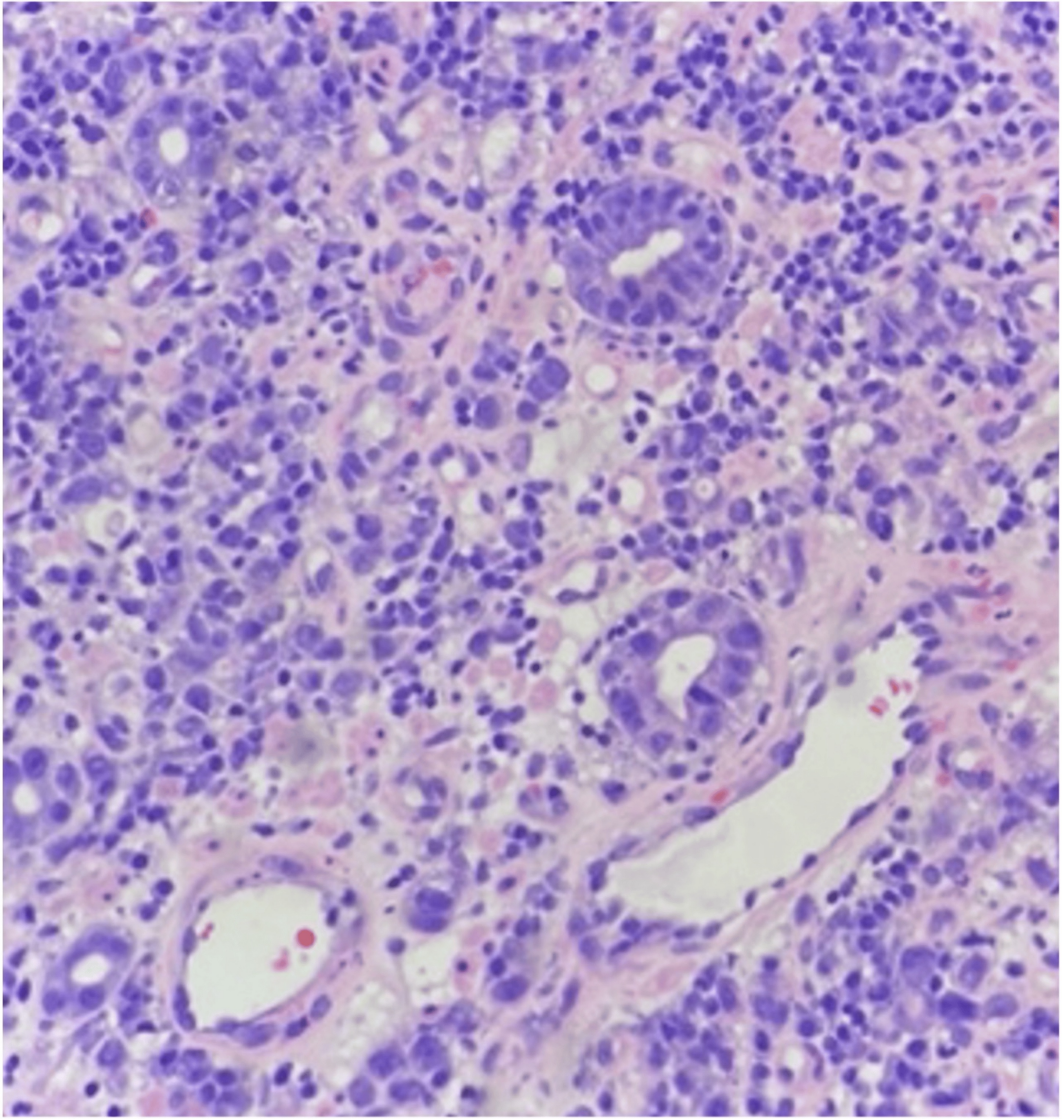
Diffuse-type gastric adenocarcinoma (haematoxylin and eosin staining, 100x magnification), demonstrating discohesive tumor cells.

A colonoscopy was conducted and was negative for polyps, masses, or changes consistent with inflammatory bowel disease. Positron emission tomography (PET) demonstrated abnormal fluorodeoxyglucose (FDG) uptake in the mid- to distal gastric body with a maximal diameter of 55 mm (Figure 2). A gastric body biopsy revealed ulcerated, poorly differentiated adenocarcinoma with focal signet ring cell features favoring poorly cohesive type (Figure 3). 

Giemsa stain was positive for *H. pylori* organisms. Genetic testing completed by NeoGenomics (NeoGenomics Laboratories, Fort Myers, FL) was negative for human epidermal growth factor receptor 2 (HER2)/neu expression. Programmed death ligand-1 (PD-L1) expression was positive with 15% staining and 2+ intensity. Mismatch repair genes were intact. The patient was initiated on a neoadjuvant chemotherapy regimen consisting of docetaxel, cisplatin, and 5-fluorouracil (DCF) prior to planned total gastrectomy.

## Discussion

EOGC rates are rising, particularly in low-incidence countries in Europe, Oceania, and the Americas. Key risk factors of GC include family history, smoking, obesity, gastroesophageal reflux disease (GERD), high salt intake, alcohol consumption, Epstein-Barr virus, and *H. pylori* infection [5]. While *H. pylori* is linked to severe gastric disease and about 3% of infections lead to non-cardia GC, its prevalence has declined globally, indicating other factors in tumor development [6]. In a nationwide population study, alcohol consumption and smoking increased the risk of EOGC in men, but not in women [7]. Proton pump inhibitors (PPIs), commonly used for GERD, are debated concerning their role in gastric carcinogenesis. Early observational studies suggest they may increase the risk of GC, atrophy, and *H. pylori *resistance [8,9]. Recent studies have revealed a significant role of gut microbiome dysbiosis and distinct genomic alterations in the development of EOGC.

Through microbiota sequencing, evidence has demonstrated elevated levels of oral microbiome bacteria in cardia and non-cardia GC cases [10]. In various meta-analyses investigating the association between gastric disease and the microbiome, *Streptococcus anginosus* (*S. anginosus*) has emerged as a consistently enriched bacterium following *H. pylori* eradication, a finding supported by level 1 evidence [11,12]. Generally found in the oral cavity, this bacterium cannot survive at a pH lower than five, and randomized control trials have demonstrated translocation into the gut with PPI use [13]. In a recent article published by Cell Host and Microbe, Fu et al. showed that *S. anginosus* can activate pro-oncogenic pathways, impair epithelial membrane integrity, and induce inflammation through the ANAX2 host receptor [14]. Their findings highlight a second bacterium or “second hit” in the ‘*H. pylori* initiation-non-*H. pylori* acceleration’ cascade. The role of probiotics remains unclear following antibiotic treatment, but supplementation with *Bifidobacterium *and *Lactobacillus* may partially assist in restoring post-eradication microbial dysbiosis [15].

The Lauren classification, first described in 1965, identifies two types of gastric adenocarcinoma: intestinal and diffuse. As in this case, diffuse gastric adenocarcinoma is marked by poorly differentiated cells with or without signet ring cells that can infiltrate the gastric wall, causing linitis plastica. Unlike the intestinal type, the diffuse type is often genomically stable, and other genetic factors are thought to contribute to EOGC due to limited environmental carcinogen exposure [16]. Tumor biology research has reformed the standard of care for a broad and expanding range of cancers. A number of surface proteins, including the PD-L1 protein and HER2, as well as intracellular deficient mismatch repair (dMMR) proteins, have potential prognostic and predictive implications, although very nuanced and cancer-specific. Conventional treatment for HER2-negative advanced GC is anti-PD-L1-based therapy. The expression of PD-L1 alone has shown limited reliability for predicting immunotherapy effectiveness in GC, particularly in low mutational burden tumors [17]. This is supported by Pietrantonio et al.’s meta-analysis of randomized clinical trials, including KEYNOTE-062, CheckMate-649, JAVELIN Gastric 100, and KEYSTONE-061, demonstrating worse survival outcomes in patients with low microsatellite instability treated with anti-PD-L1-based treatment compared to patients with high microsatellite instability [18]. Aside from targetable proteins and genomic integrity, epigenomic studies provide further insight into EOGC tumor regulation and behavior. 

Genome-wide analysis has identified single-nucleotide polymorphisms (SNPs) in XRCC genes and alterations in BANP, MUC5B, RHOA, ARID1A, and TGFBR1 [19,20]. Ge et al. recruited 12 GC patients to show a significant difference in genome-wide methylation expression between patients with EOGC and late-onset GC. The study speculated that hypermethylation of Cg11037477, a promoter region of the eukaryotic translation initiation factor 4E (EIF4E), may lead to deregulation of the well-established oncologic PI3K-AKT pathway. A significant association was found between age at diagnosis and expression of EIF4E with hypermethylation of the promoter region (p<0.05). In addition, the down-expression of EIF4E was associated with poor survival [4]. The patient we describe has an SNP (Gln77Gln) in the XRCC2 gene, which is involved in DNA double-strand repair. As with CDH1, the relevance of XRCC2 with EOGC is not readily established or identified as a hereditary factor. These studies suggest that early-onset GCs show distinct genomic and epigenomic features compared to those that arise later in life.

## Conclusions

The prevalence of GC in individuals under 50 years old is increasing, and healthcare practitioners should closely monitor symptoms in this population. Testing for *H. pylori*, initiating eradication therapy with confirmation of eradication, and practicing judicious use of PPIs may offer clinical benefit, particularly in individuals under 50 with dyspeptic symptoms. Research on the gut microbiome has demonstrated the causality of *S. anginosus* in gastric tumorigenesis and established a mechanism for oncogenesis. Currently, no guidelines support interval screening with EGD for detecting EOGC; however, genomic testing may help identify individuals at higher risk and justify earlier screening. The risk factors for GC are well established, but more studies are needed to differentiate between early-onset and later-onset GC. Overall, further research into developing primary and secondary prevention strategies is crucial for reducing the burden of GC. This case highlights the importance of considering various risk factors to improve risk stratification and enhance surveillance efforts.
